# The Surgical Anatomy of the Jowl and the Mandibular Ligament Reassessed

**DOI:** 10.1007/s00266-022-02996-3

**Published:** 2022-09-01

**Authors:** Lennert Minelli, Hun-Mu Yang, Berend van der Lei, Bryan Mendelson

**Affiliations:** 1 Melbourne Advanced Facial Anatomy Course (MAFAC), Australasian Society of Aesthetic Plastic Surgeons (ASAPS), PO Box 592, Toorak, VIC 3142 Australia; 2 Medical Engineering Research Facility (MERF), Queensland University of Technology (QUT), Brisbane, QLD Australia; 3 Department of Anatomy and Physiology, School of Biomedical Sciences, The University of Melbourne, Melbourne, VIC Australia; 4 Department of Plastic Surgery, University Medical Centre Groningen, University of Groningen, Groningen, The Netherlands; 5 Department of Anatomy, Yonsei University College of Medicine, Seodaemun-gu, Seoul, South Korea

**Keywords:** Facial retaining ligaments, Mandible, Jowl, Labiomandibular fold, Platysma, Aging

## Abstract

**Introduction:**

A visible jowl is a reason patients consider lower facial rejuvenation surgery. The anatomical changes that lead to formation of the jowl remain unclear. The aim of this study was to elucidate the anatomy of the jowl, the mandibular ligament and the labiomandibular crease, and their relationship with the marginal mandibular branch of the facial nerve.

**Materials and Methods:**

Forty-nine cadaver heads were studied (16 embalmed, 33 fresh, mean age 75 years). Following preliminary dissections and macro-sectioning, a series of standardized layered dissections were performed, complemented by histology, sheet plastination and micro-CT.

**Results:**

The jowl forms in the subcutaneous layer where it overlies the posterior part of the mandibular ligament. The mandibular ligament proper exists only in the deep, sub-platysma plane, formed by the combined muscular attachment to the mandible of the specific lower lip depressor muscles and the platysma. The mandibular ligament does not have a definitive subcutaneous component. The labiomandibular crease inferior to the oral commissure marks the posterior extent of the fixed dermal attachment of depressor anguli oris.

**Conclusion:**

Jowls develop as a consequence of aging changes on the functional adaptions of the mouth in humans. To accommodate wide jaw opening with a narrowed commissure requires hypermobility of the tissues overlying the mandible immediately lateral to the level of the oral commissure. This hypermobility over the mandibular attachment of the lower lip depressor muscles occurs entirely in the subcutaneous layer to allow the mandible to move largely independent from the skin. The short, elastic subcutaneous connective tissue, which allows this exceptional mobility without laxity in youth, lengthens with aging, resulting in laxity. The development of subcutaneous and dermal redundancy constitutes the jowl in this location.

**Level of Evidence IV:**

"This journal requires that authors assign a level of evidence to each article. For a full description of these Evidence-Based Medicine ratings, please refer to the Table of Contents or the online Instructions to Authors www.springer.com/00266."

**Graphical Abstract:**

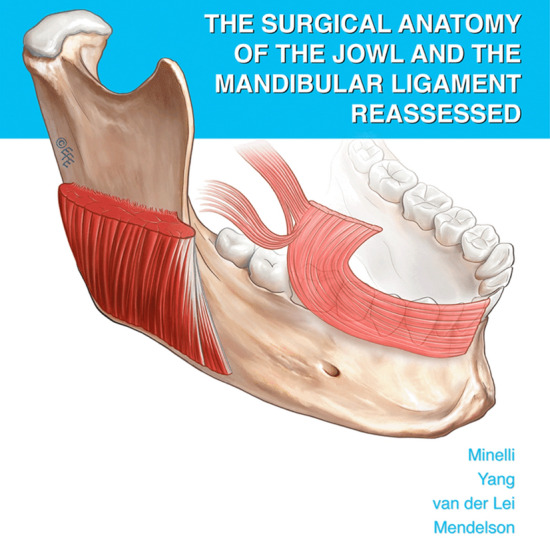

**Supplementary Information:**

The online version contains supplementary material available at 10.1007/s00266-022-02996-3.

## Introduction

“*Follow? Nay, I'll go with thee, cheek by jowl.*”

–*A Midsommer Nights Dreame, William Shakespeare, 1596*

The jowl is a key stigmatizing feature of the aging human face and a contributing reason why lower facial rejuvenation is considered. However, the aging changes in the underlying anatomy that lead to the appearance of the jowl and its exact anatomical borders remain unclear. The jowl is currently regarded as sagging of redundant facial tissue that is bordered anteriorly by the mandibular ligament at the labiomandibular crease. Release of the mandibular ligament is indicated in certain situations to obtain full correction of the jowl during facelift surgery.

**Fig. 1:**
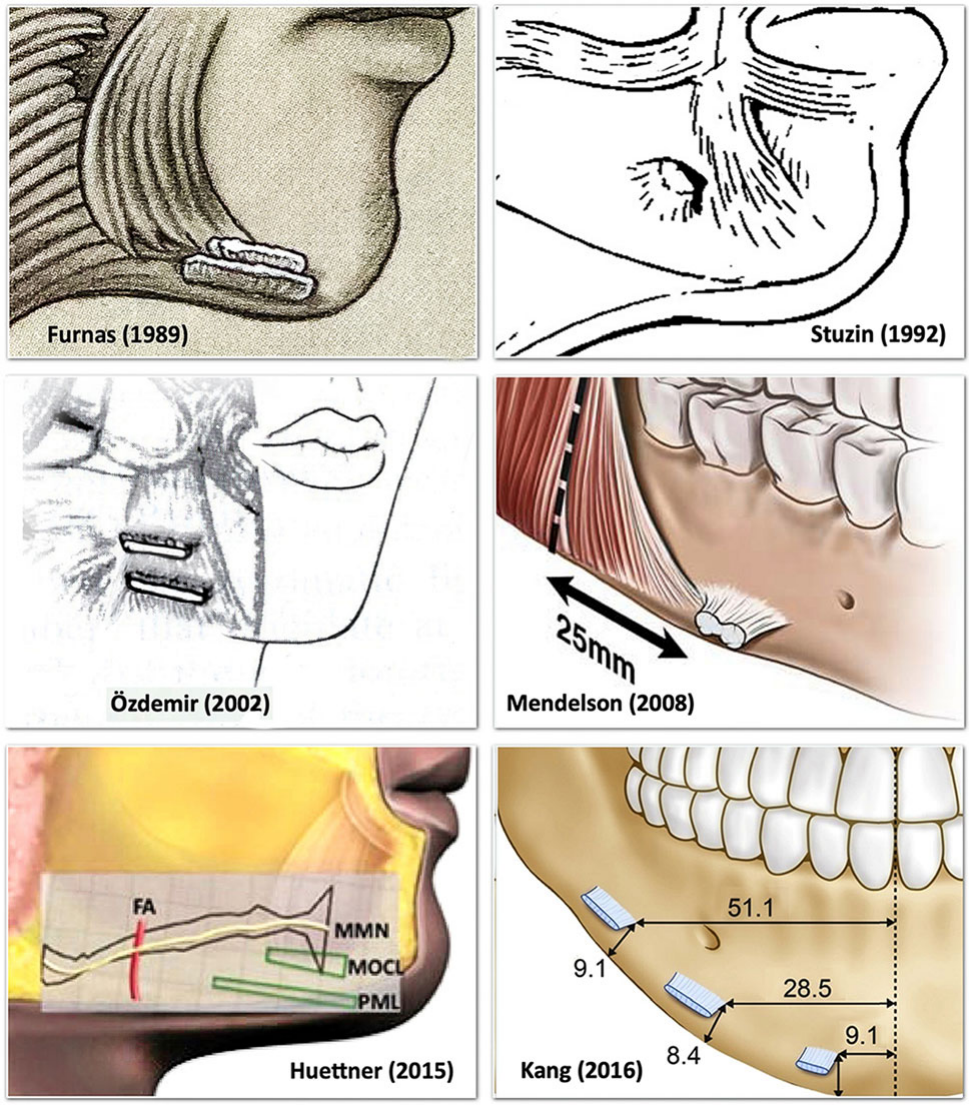
This illustration combines the different historical descriptions of the mandibular ligament. Furnas [3] introduced the mandibular ligament as “a linear series of parallel fibres along the anterior third of the mandible which interdigitate among the muscle fibres of the platysma and triangularis along their line of attachment to the mandible”. Stuzin et al. [4] described the mandibular ligaments as osteocutaneous which “securely fix the parasymphysial dermis to the underlying mandible” and illustrated it as a smaller stout ligament. Özdemir et al. [5] reported two mandibular ligaments with mean widths of 22−32 mm. Mendelson et al. [1] reported it in the sub-platysmal plane: “the mandibular ligament is located immediately in front of the masseter’s anterior border”. Huettner et al. [7] described two ligaments in the subcutaneous plane: the mandibular osteocutaneous ligament (MOCL) with a mean width of 13 mm, and the platysma mandibular ligament (PML) with a mean width of 22 mm. Kang et al. [8] described two mandibular ligaments and one mental ligament in the sub-platysmal plane. The platysma, DLI and DAO mandibular attachments were not mentioned, nor a subcutaneous extension of these ligaments Reproduced with permission from Wolters Kluwer Health, Springer Nature, Oxford University Press and Elsevier

The presence of a mandibular ligament was first described by Furnas in his 1989 classic paper, *The Retaining Ligaments of the Cheek*, along with his original description of the zygomatic ligament. Given the similarity of their function as retaining ligaments, there has been a natural assumption that these ligaments are anatomically similar. Since its description, the role of the mandibular ligament has been considered central in the development of the jowl with aging. To date, there have been two different explanations regarding the place of the ligament in the pathophysiology of the jowl. Mendelson et al. described the jowl as being in the deep (sub-platysma) plane, whereas Reece et al. described its presence in the subcutaneous plane (supra-platysmal).[1, 2] The mandibular ligament itself has also been reported with considerable variation regarding its position and extent (Fig. 1).[1, 3–8] Recent studies have described the mandibular ligament to extend up to 65 mm from the midline and 45 mm from the gonial angle, which locates it much more posterior than at the anterior border of the jowl.[8, 9] The labiomandibular crease was demonstrated not to be formed by a ligament but by the direct insertion of the lower lip muscles into the dermis, like the nasolabial crease in the upper lip.[10, 11]

This lack of an agreed understanding has hampered confident understanding of this region during facelift surgery. Therefore, this anatomical study was undertaken to elucidate the true nature of the jowl, including its bordering structures, the labiomandibular crease and the exact location of the mandibular ligament and its relationship with the marginal mandibular branch of the facial nerve (MMN).

## Materials and Methods

Ethical approval for the project was granted by the Human Ethics Advisory Groups of the University of Melbourne for the exploratory study, the Queensland University of Technology for the definitive study including histology and plastination, and the Yonsei University College of Medicine for the micro-CT study (Project numbers 14243, LR2021-4306-4761, and YSAEC22-004).

First, an exploratory study was performed on 21 cadavers, fifteen embalmed and six fresh frozen (male = 10; female = 11; mean age = 76 years) to determine how to most effectively study this area of complex anatomy. This involved layered dissections, facelift dissections and macro-sectioning in various planes.

Based on this feasibility study, a definitive study was conducted using standardized dissections on 14 cadavers, one embalmed and thirteen fresh (non-frozen) (male = 8; female = 6; mean age = 80 years). For the dissection, following markings and measurements, a skin incision was made over the clavicles through the subcutaneous fat to the platysma. Then, a sweeping motion of a no. 10 blade was used along the superficial surface of the platysma to define areas of increased attachment of the skin to the platysma. The distance from the midline to such attachments was measured. Dissection was then performed at the deep surface of the platysma to determine the deep part of the mandibular ligament as well as the attachments of the platysma, DLI and DAO to the mandible.

Finally, objective technical investigations were used to complement the dissection findings:

1. Histology of full-thickness macro-sections of the jowl region was studied in thirteen samples of six cadavers (male = 3; female = 3; mean age = 77 years).
2. Sheet plastination of the head and neck of ten fresh cadavers was processed by *von Hagens Plastination* in the axial, sagittal and coronal planes using their latest technique (n = 10; male = 4; female = 6; mean age: 67 years).[12]
3. High-resolution micro-CT of the mandibular area was performed on two hemifaces of one cadaver to confirm the anatomy in a three-dimensional way (male, 67 years).[13]

## Results

The anthropometric results of the mandibular area are shown in Table 1. The series of standardized anatomical dissections demonstrated that the jowl is a localized area of redundant skin and subcutaneous fat, that the anterior border of the jowl and labiomandibular crease is not defined by a specific osteocutaneous ligament but by there being a change in subcutaneous connective tissue organization, and that the deep part of the mandibular ligament is the unique combined attachment of the platysma, DLI and DAO into the mandible which actually underlies the jowl partly, not borders it anteriorly. The sheet plastination confirmed these observations, while the histology and micro-CT results demonstrated the true anatomical nature of the jowls.

**Table 1:** Measurements on the jowl, mandibular ligament and relevant structures obtained from a standardized series of dissections on twelve fresh (non-frozen) cadavers
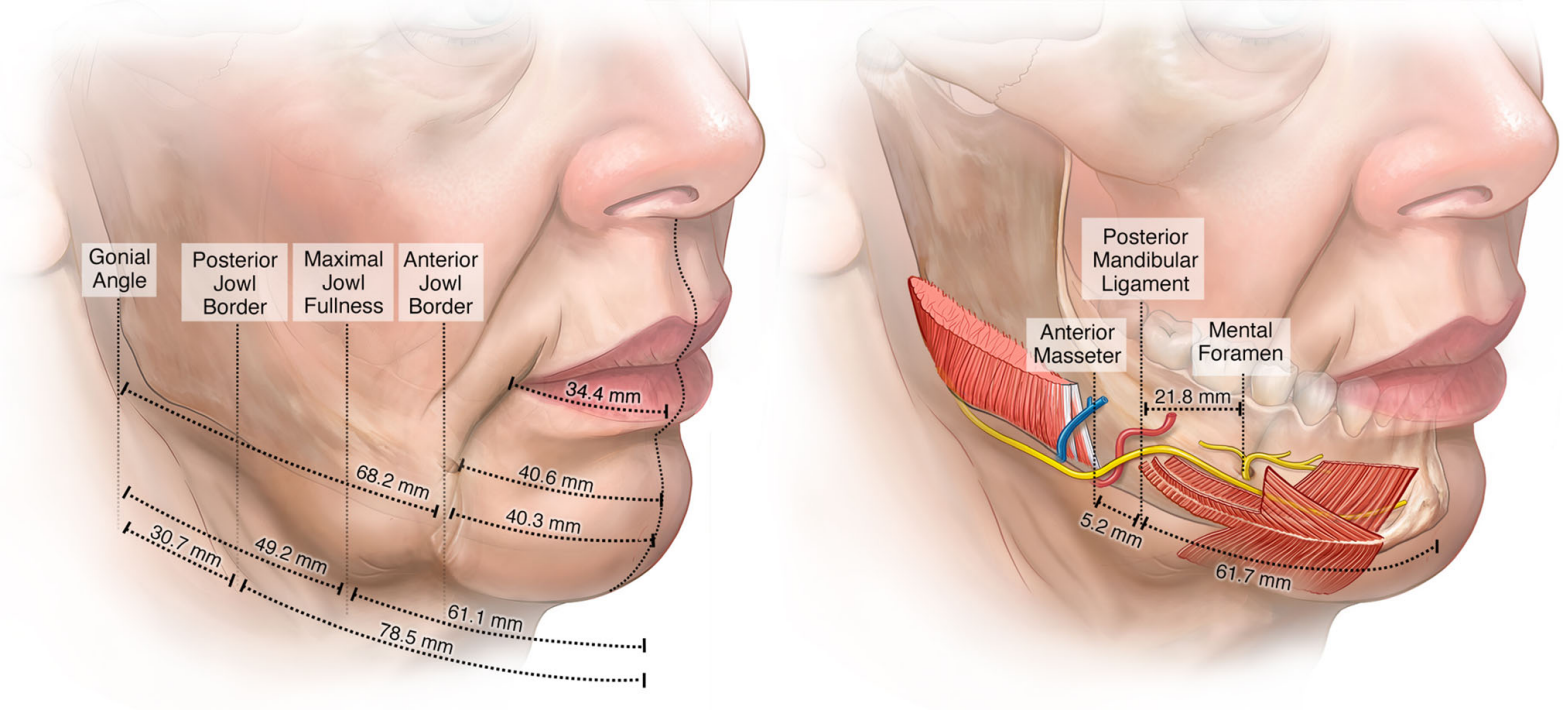

	Mean (mm)	SD (mm)	Range (mm)
Midline—Mental Tubercle	13.0	3.8	9 − 19
Midline—Corner of Mouth	34.4	3.9	29 – 40
Midline—Marionette Crease	40.6	4.1	35 – 47
Midline—Posterior extent of Mandibular Ligament	61.7	4.9	52 – 68
Midline—Gonial Angle	113.2	7.4	101 – 123
Posterior extent of Mandibular Ligament—Masseter Muscle	5.2	5.0	−6 – 10
Posterior extent of Mandibular Ligament—Mental Foramen	21.8	2.3	19 – 26
Posterior extent of Mandibular Ligament—MMN (n. VII)	1.5	0.5	1 – 2
Midline—Anterior Jowl Border	40.3	3.6	33 – 44
Midline—Maximal Jowl Fullness	61.1	6.9	48 – 72
Midline—Posterior Jowl Border	78.5	8.3	67 – 98
Gonial Angle—Anterior Jowl Border	68.2	11.3	48 – 91
Gonial Angle—Maximal Jowl Fullness	49.2	9.4	33 – 69
Gonial Angle—Posterior Jowl Border	30.7	8.0	19 – 50

### The Jowl

The jowl is situated entirely in the supra-platysmal plane, as redundant subcutaneous tissue with overlying skin. The maximal jowl fullness corresponds with the area over the posterior end of the mandibular ligament. This area exhibits the longest length of the retinacula cutis fibres in the subcutaneous plane on histology and micro-CT (see further: “Superficial part of the Mandibular Ligament”). The sub-platysmal structures do not add to the jowl volume but to adjacent prominences: the buccal fat pad bulges superior to the jowl, and the submandibular gland, when prominent, is inferior to the jowl

### Deep Part of the Mandibular Ligament

**Fig. 2:**
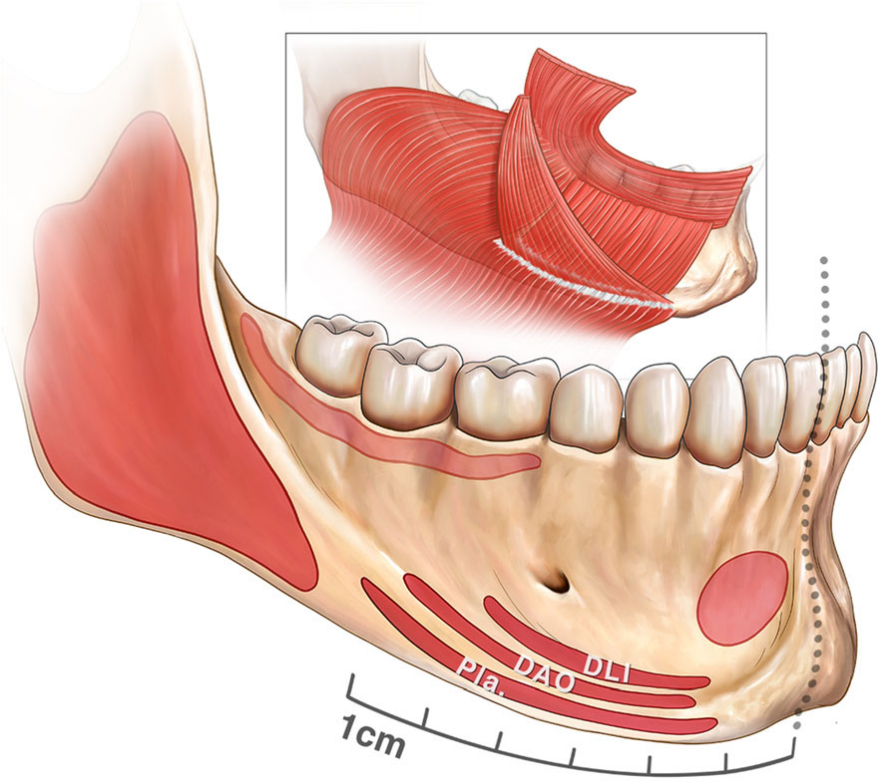
The deep part of the mandibular ligament is the combined mandibular attachment of the platysma, depressor labii inferioris (DLI) and the depressor anguli oris (DAO). It has a specific organization with the platysma attaching most caudal, the DLI attaching most cephalad and the DAO attaching in the middle. The posterior part of the platysma also inserts directly into the buccinator and the modiolus. The middle part of the platysma “disappears” deep to the DAO to “reappear” at its medial border and insert into the lower lip dermis and orbicularis oris muscle. This part was previously called the “depressor labii lateralis” by Le Louarn.[23] The fixed anterior part of the platysma inserts into the mandible as part of the mandibular ligament but then continues further to the lower lip under the name “depressor labii inferioris”, which is embryologically and evolutionary part of the platysma muscle. When dissecting in the deep plane, it is the posterior end of the platysma attachment which can be felt as the ligament when palpating the flap anteriorly

**Fig. 3:**
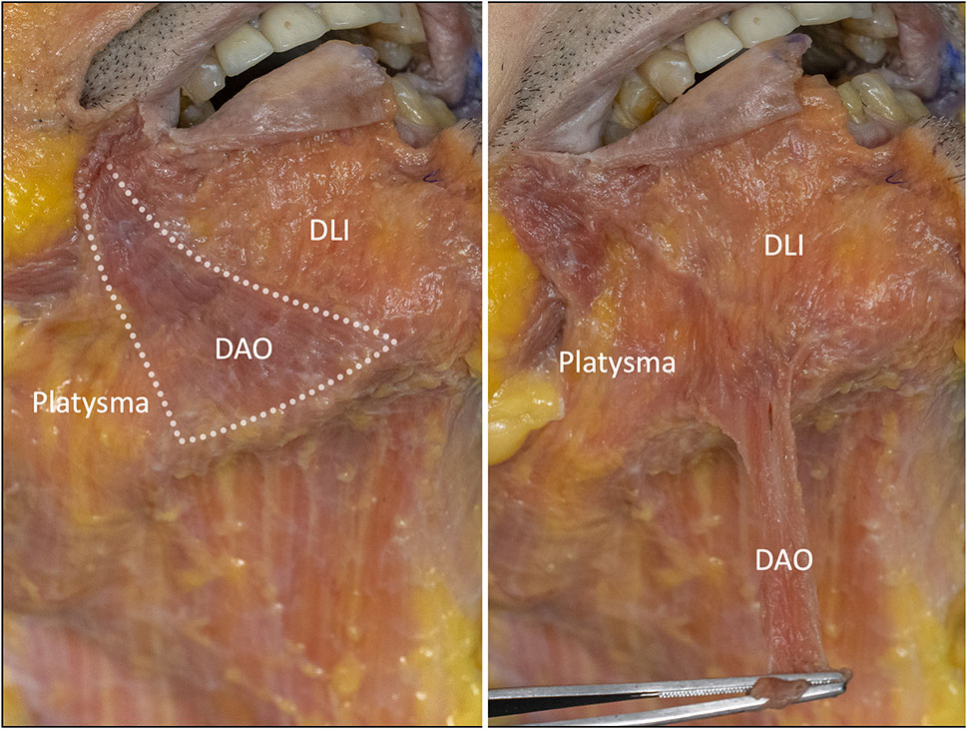
This right side of a fresh cadaver demonstrates the lower lip depressors. Removal of the depressor anguli oris (DAO) demonstrates the continuity of the platysma as the depressor labii inferioris (DLI), being a continuation of the same muscle

In the sub-platysmal plane are the attachments of the platysma, DLI and DAO into the anterior third of the mandible. These three muscles attach to the mandible in a particular and consistent manner, with the platysma most caudal, the DLI attachment most cephalad, and the DAO in between these two muscles (Fig. 2). Posterior to the mandibular ligament, the platysma is loosely “connected” to the mandible by areolar connective tissue which allows significant mobility of the platysma over the mandible (Video 1). The muscle fibres of this posterior part of the platysma continue over the mandible, without skeletal attachment, to insert directly into the buccinator and the orbicularis oris muscle at the modiolus. The DLI was confirmed to be part of the platysma layer, being entirely continuous with it (including the same direction of muscle fibres) and with no overlap being present between these two muscles. Removal of the DAO, which overlies both muscles, demonstrated this continuity (Fig. 3, Video 2).

The only firm attachment of the musculoaponeurotic layer (Layer 3) to the mandible was that of these three muscles, which we considered to be the “mandibular ligament” of the deep plane, more for descriptive purposes**.**

**Fig. 4:**
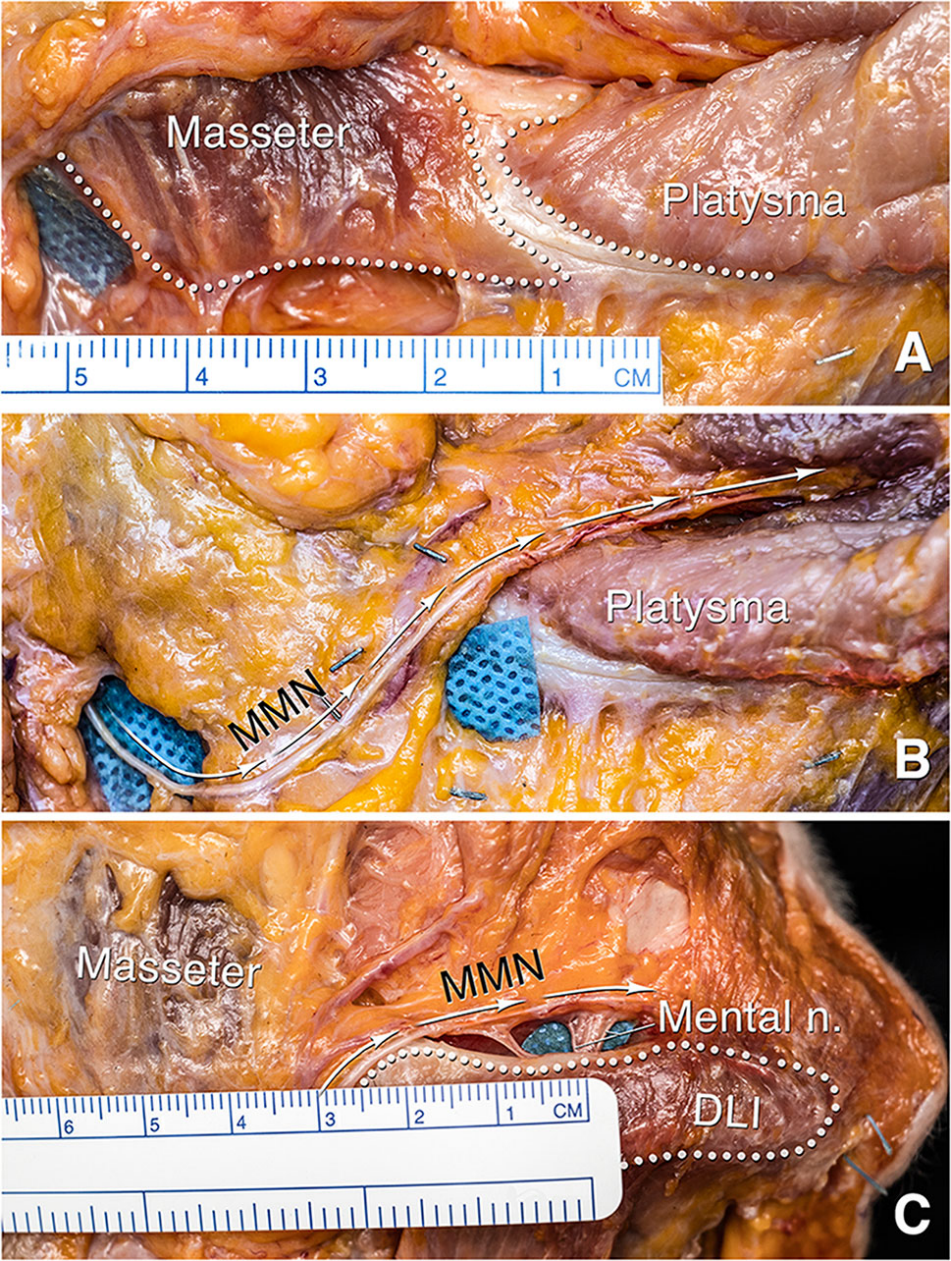
Fresh cadaver dissections of the right mandible. **A** The mean distance from the posterior end of the platysma attachment to the masseter is 5.2 mm (SD = 5.0 mm), but in some cases, such as the one seen here, the two muscle attachments “overlap”, with the platysma, DLI and DAO taking origin superior and the masseter taking origin inferior on the mandible. **B** Repositioning the deep fascia over the mandible demonstrates there is a wafer of fat overlying the mandible and masseter posterior to the mandibular ligament (also clear in C.). Coursing vertically through this fat are the facial vein posteriorly and the facial artery anteriorly with the marginal mandibular nerve (MMN) crossing both vessels superficially. The fat over the nerve has been removed to enhance visualization. The main branch of the MMN is always in close relationship with the mandibular ligament, passing it at only 1 - 2 mm to then continue forward, cephalad to the ligament towards the mentalis, still deep to the platysma, DLI and DAO. **C** With the upper part of the platysma, DLI and DAO flipped over to reveal what is behind, the continuation of the main branch of the MMN is visualized passing towards the mentalis muscle. The mental nerve is seen exiting the mental foramen at approximately 22 mm from the end of the mandibular ligament

The mean extent of this mandibular ligament starting from the midline was 63.1 mm in men and 59.3 mm in women, corresponding with the location of maximal jowl fullness, not the anterior border of the jowl (Table 1). The mean distance from the posterior end of the mandibular ligament to the masseter muscle attachment was a mere 5.2 mm. Moreover, in two cases there was no space between the two, and in one case, they even overlapped with the masseter attaching inferior and the platysma, DLI and DAO attaching superior on the mandible (Fig. 4.A).

### Superficial Part of the Mandibular Ligament

**Fig. 5:**
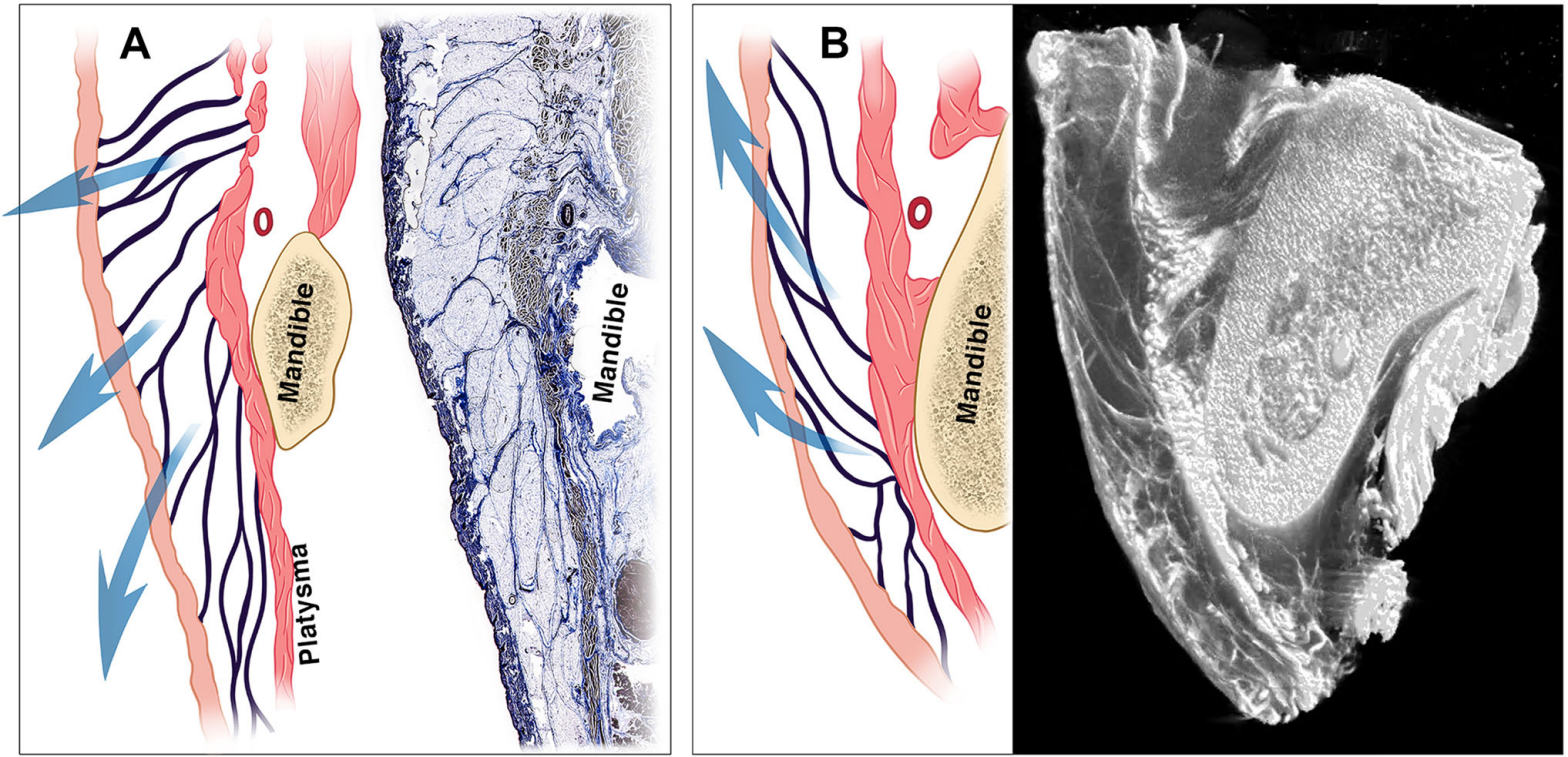
Overview of the subcutaneous soft tissue organization at the level of the jowl approximately at the most posterior end of the mandibular ligament. Typical (**A**) histology with the mouth closed and (**B**) micro-CT with the mouth open, of the jowl demonstrates the enormous mobility of the skin and subcutaneous fat in this area. Instead of a stout ligament in the subcutaneous layer, the retinacula cutis connecting the skin to the muscles over the mandibular ligament are longer than in the other supra-platysmal areas. This allows the skin to glide over the mandibular ligament when opening the mouth. Note how the retinacula cutis are oriented downwards with the mouth closed, but upwards with the mouth open, allowing the gliding of the mandible and muscle attachment underneath. When dissecting in the deep subcutaneous plane, the length of these retinacula cutis cannot be perceived as they are cut at their base (trunk), and they can be perceived as subcutaneous “mandibular ligaments”

In the subcutaneous plane, no significant ligaments were identified. The retinacula cutis fibres attaching the skin to the platysma are not denser/thicker in the area overlying the deep mandibular ligament than in the surrounding subcutaneous tissues. Instead of providing stout fixation, the subcutaneous retinacula cutis septa are longer in the area of the jowl than in other areas, which allows significant mobility of the skin over the fixed platysma (Fig. 5). However, when performing a deep subcutaneous dissection at the lower trunks of these retinacular fibres, the flap does *appear* more tethered to the platysma over this area than over the surrounding areas. This is not because of the presence of a strong ligament in the subcutaneous connective tissues, but because the platysma at this location is tethered by its attachment to the mandible. Whereas traction on the flap in all other areas results in *gliding* of the platysma over the deeper tissues, in contrast to traction over the deep mandibular ligament, which is met with resistance. For the untrained eye this might appear as “skin tethering” but this is, in fact specifically “muscle tethering” (Video 3).

### The Labiomandibular Fold and Crease

**Fig. 6:**
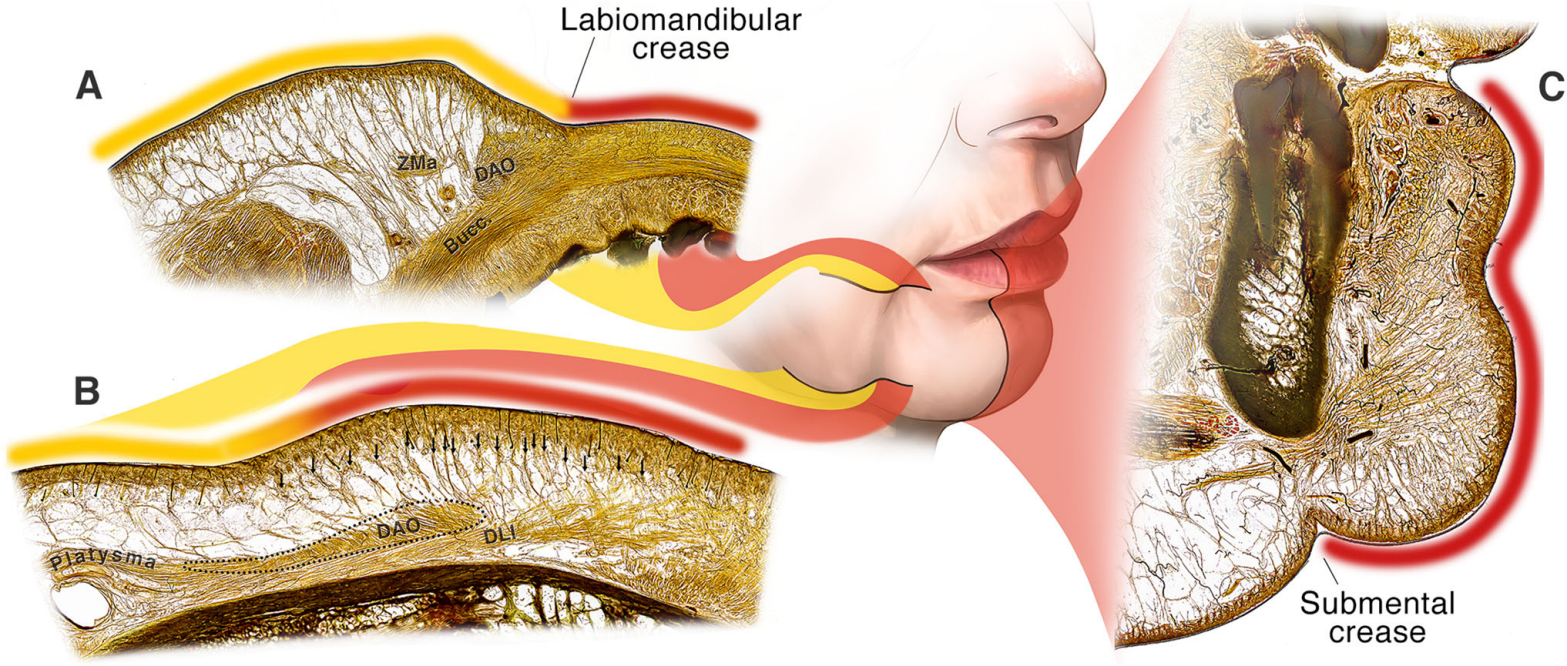
Sheet plastination sections through three planes of the chin region demonstrate the shift from loose areolar tissue in the normal cheek and neck tissues to dense adhesion of the lower lip muscles and the dermis in the perioral adhesion zone. **A** Axial section through the cephalad part of the lower lip. Observe the retinacula cutis fibres connecting the dermis to the muscle are longer in the cheek, abruptly shortening anteriorly in the perioral region over the DAO to become absent medial to the DAO. **B** Axial section through the caudal part of the lower lip demonstrates a more subtle transition and no real labiomandibular crease or fold can be pinpointed. Whereas posteriorly, the dermis is loosely connected to the muscles, this connection becomes more defined at the anterior third of the DAO, with thicker and shorter retinacula cutis. At the anterior border of the DAO, the DLI also starts inserting directly into the lower lip skin. **C** Sagittal section of the chin segment showing the relatively uniform tight attachment of the dermis that maintains the soft tissue connection with the mandible on movement. Note the abrupt change in the neck inferior to the submental crease

The labiomandibular crease represents the start of the so-called *perioral adhesion zone* where the upper and lower lip muscles insert directly into the dermis. This dermal-muscle adhesion starts at the alar base to include the lip levator muscles forming the nasolabial crease, continues over the anterior part of the modiolus and the superomedial border of the DAO forming the labiomandibular crease to finally include the transversus menti muscle forming the submental crease. The net effect of these muscles inserting into the dermis is a radical change in the subcutaneous layer (Layer 2). Whereas lateral to the crease the subcutaneous fat allows mobility of skin over the muscles, medial to the crease the skin and mimetic muscles are adherent and move as one entity. Sheet plastination confirmed this configuration (Fig. 6).

The labiomandibular fold is most defined just inferior to the modiolus, where the platysma emerges from underneath the DAO and inserts directly into the skin and into the orbicularis oris muscle (Fig. 6A). When following the crease inferiorly, it becomes less distinct, partially overlying the DAO. Here, the DLI and mentalis muscles are seen inserting into the dermis in a more diffuse way (Fig. 6B).

### Marginal Mandibular Nerve

A wafer of deep fat is consistently seen overlying the mandible between the bulk of the masseter muscle and the mandibular ligament (Fig. 4B). This fat also covers the anterior extension of the masseter muscle, which is often overlooked. The following structures were consistently visualized within this wafer of fat: the facial vein posteriorly, the facial artery anteriorly, and the MMN crossing both vessels superficially. The main branch of the MMN was always in intimate relation with the mandibular ligament, passing 1 - 2 mm from it to continue cephalad to the ligament still deep to the platysma, DLI and DAO (Fig. 4C, Video 2).

## Discussion

This unique anatomical study, with initial exploratory dissections and macro-sectioning, a series of standardized layered dissections complemented by histology, sheet plastination and micro-CT has clearly clarified the following structures: the jowl is redundant subcutaneous tissue with its overlying skin, not bordered anteriorly by an osteocutaneous mandibular ligament but by the perioral adhesion zone of direct lower lip muscle insertion into the dermis. The mandibular ligament proper is present only in the deep (sub-platysmal) plane and extends underneath the jowl up to the level of the *maximal jowl fullness*, not to the anterior border of the jowl. The MMN branch passes the mandibular ligament in a much more intimate position than was previously suggested.

### Functional Anatomy

The entire mandibular anatomy in the jowl area reflects the complex functional requirements necessary for opening the mouth, mastication and mimetic activity. All these actions require slightly different planes of mobility and points of attachments, which explain the effects seen with aging in this region, i.e. the jowl.

#### Speech and Mimicry

**Fig. 7:**
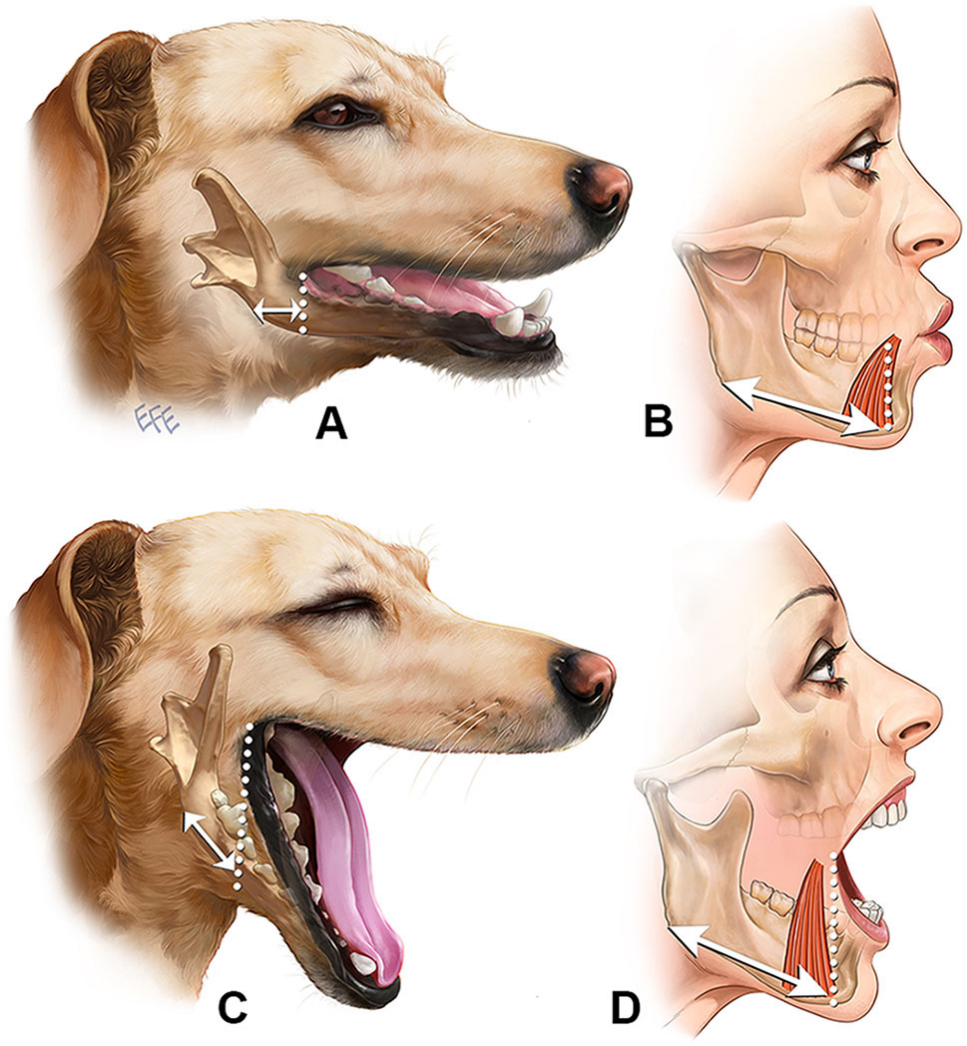
This illustration comparing the human to the dog illustrates how much soft tissue needs to glide over the mandible posterior to the oral commissure for the jaw to open. The human has a very narrow mouth combined with a broad mimetic muscle attachment (platysma, DLI, DAO). This combination requires for skin to glide over this area (or rather the mandible and muscle attachments to glide under the skin), eventually creating the jowl

In contrast to other mammalian species, humans have a relatively narrow mouth and commissures to allow complex lip movement (Fig. 7). In addition, humans have mimetic muscles of the lower lip (*depressors*), which attach in a relative broad area onto the mandible, being approximately 3 cm posterior to a vertical line though the oral commissure.

#### Opening the Mouth

**Fig. 8:**
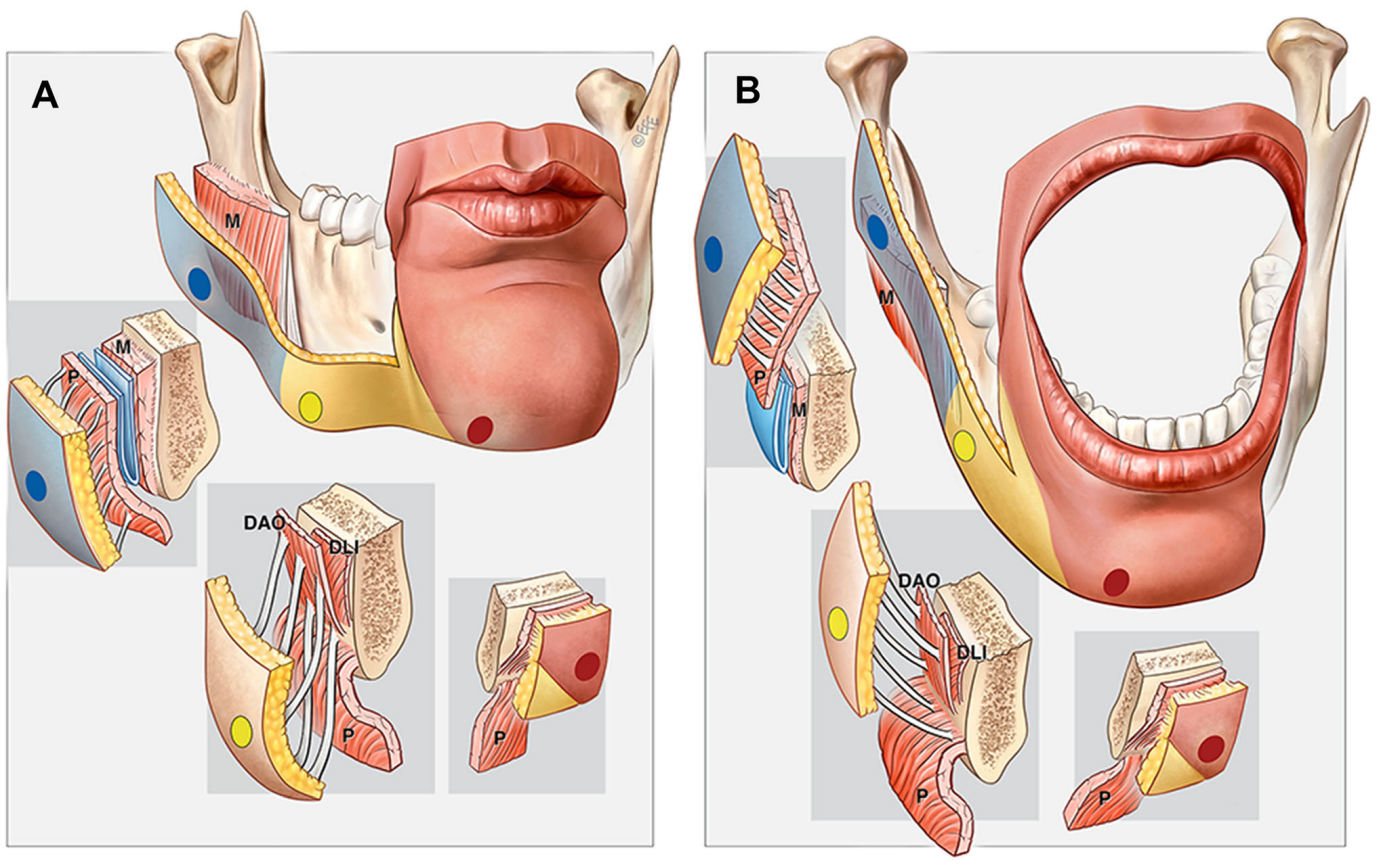
This illustration demonstrates how the different areas across the mandible react to opening of the mouth. At the premasseter space (blue), opening the mouth results in gliding of the platysma over the masseter without need for additional skin gliding. At the mandibular ligament (yellow), opening the mouth requires the skin to glide over the mandible-muscle complex at the common mandibular attachment of the platysma, DLI and DAO. At the perioral adhesion zone (red), opening the mouth results in en-bloc movement of the mandible, lower lip muscles and the skin, maintaining constant relationships

The mean opening of the mouth (and thus lowering of the mandible) in the Caucasian population is approximately 5 cm (range 3.7 – 6.7 cm).[14] While the chin and lower lip skin simply follows the mandible, the position of the oral commissure and especially the cheek does not lower by the same amount. Lowering of the mandible therefore needs to be largely independent from the soft tissues in order to prevent the cheek to be pulled down during mouth opening. In humans, the area where the mandible must glide independent from the soft tissues is much larger than in most other species due to the narrow mouth (Fig. 7). Furthermore, the broad mandibular attachment of mimetic muscles in humans causes these muscle origins to be dragged along when lowering the mandible. As the lip depressors elongate, the overlying skin which lacks this intrinsic stretching capability needs to allow the underlying muscles and mandible to glide. This need for gliding requires a specialized subcutaneous connective tissue arrangement in this area, not a firm immobile osteocutaneous ligament (Fig. 8).

### Development of Jowls

The functional anatomy may explain how the formation of the jowl is related to the necessity for skin gliding. Whereas in youth, the subcutaneous connective tissue is elastic and shorter, in due time with aging, it loses this elasticity and lengthens, resulting in secondary subcutaneous laxity that constitutes the jowl. The jowl is maximal overlying the most posterior part of the mandibular ligament, as most gliding of the skin over the muscles occurs here. Towards the chin, the necessity for skin to glide lessens and thus waning of the jowl anteriorly. Posterior to the mandibular ligament, gliding of the mandible occurs mostly in the sub-platysmal plane (premasseter space), reducing the necessity for skin to glide and thus waning of the jowl posteriorly (Video 1). No individual gliding of the skin over the muscle is possible nor necessary over the area of the perioral adhesion zone.

### The Mandibular Ligament

Since its first description in 1989, most clinicians were satisfied with the concept that an osteocutaneous ligament must mark the anterior border of the jowl. Since then, research regarding this idea has focused on uncovering the true nature and location of this ligament. However, the presence of an osteocutaneous ligament at this location would impair mouth opening by directly tethering the mandible to the midcheek skin and via the skin to the zygoma.

Our study provides clear evidence that the mandibular ligament exists only in the deep plane (sub-platysmal) as the muscular attachment of the platysma, DLI and DAO to the mandible, not in the subcutaneous plane. Interestingly, this pattern of attachment of these muscles to the mandible had already been described in classic anatomical textbooks.[15, 16] In the subcutaneous layer, the long retinacula cutis provided mobility rather than fixation. Interestingly, this organization is remarkably similar to the mandibular septum described by Reece, Pessa and Rohrich.[17]

Skin tethering as seen during a subcutaneous dissection at this location may mimic a subcutaneous mandibular ligament but was clearly demonstrated to be nothing more than a reflection of the underlying muscle tethering to the mandible. The lack of a true osteocutaneous ligament explains the large variety of descriptions of subcutaneous mandibular ligaments by previous authors who were looking to define a subcutaneous ligament in this area. This understanding also explains why the mandibular ligament was consistently discovered much further posterior than the anterior border of the jowl: it was identified along the entire fixed platysma.[9]

### The Labiomandibular Fold and Crease

Our anatomical study confirms previous reports that the labiomandibular crease is not caused by an osteocutaneous ligament but simply marks the start of the insertion of the lower lip muscles into the dermis similar to the nasolabial crease of the upper lip (i.e. perioral adhesion zone).[10, 11, 18–20] In addition to the report of Pessa et al. describing DAO inserting into the dermis superiorly in the crease, this study showed that (from superior to inferior) platysma, DLI and mentalis add to this dermal insertion along the superomedial border of the DAO.[10] Whereas the crease is very well defined superiorly, the inferior extent is not.

### Marginal Mandibular Nerve

The standardized set of anatomical dissections in our study reliably located one branch of the MMN in much closer proximity to the mandibular ligament than previously suggested. Whereas Huettner et al. described its presence at a mean distance of 9.7 mm from the subcutaneous “mandibular ligament”, we found it consistently present in close proximity, only 1-2 mm from the deep mandibular ligament.[7] The explanation for the disparity in the findings from Huettner et al. is understandably due to the different depth of dissecting the mandibular ligament. The ligament we isolated and consider to be the real mandibular ligament is in the deep plane, whereas Huettner’s plane of dissection was in the subcutaneous plane. As the MMN runs in the deep plane (sub-platysmal), to visualize the MMN required the removal of a strip of overlying platysma, DLI and DAO to then measure the distance from the “deep” MMN to the “subcutaneous” mandibular ligament.

### Implications for Rhytidectomy

Correction of the jowl by a deep-plane, sub-platysmal lower facelift technique might seem counterintuitive in the presence of the mandibular attachment of the platysma impairing lifting the platysma and overlying anterior part of the jowl. However, the laxity of the elastic platysma muscle sheet allows significant lifting despite its mandibular attachment, with the overlying tissues being redraped and the jowl effaced by this manoeuvre. The improvement of the jowl obtained using the deep-plane (sub-platysma) lower facelift technique thus results from tightening the laxity of the elastic platysma superior to its attachment. This lift is transmitted through the retinacula cutis to reduce the overlying dermal laxity, which flattens the jowl to a significant degree.

The release of the true (deep) mandibular ligament can only be done through a subperiosteal release of the broad ligament, e.g. through a submental incision. This is because releasing the mandibular ligament in the deep plane carries too much risk due to the close proximity of the MMN and is therefore not recommended. Release of the “mandibular ligament” in the subcutaneous plane is simply releasing the connection of the dermis of the anterior jowl to the musculoligamentous attachment of the platysma, DLI and DAO. Unlike the idea that this happens at the anterior border of the jowl, this actually occurs along the anterior half of the jowl which explains why this manoeuvre is successful: it was never clear before how it could be beneficial to release a ligament anterior to the jowl when the redraping is posterior. Instead, what is actually released is the tethering of the skin over the mandibular ligament at the anterior half of the jowl. This release may be indicated (1) to tighten submental laxity from the lateral approach or (2) to release a significant residual skin crease in this area, using the submental approach.[21, 22]

### Limitations of the Study

The cadavers studied were all from a similar age (range 56 – 97). As the jowl appears and progresses over time, it would be ideal to study the development of the jowl throughout aging done on groups of cadavers of different ages. Sheet plastination was only obtained in three planes, axial, sagittal and coronal. Ideally, a plane perpendicular to the mandible would be used. Histological investigations required the removal of the mandible and after laying the sample flat on a cardboard for fixing. Ideally, the anatomy would be investigated with the tissues undisturbed, e.g. with decalcification of the mandible in situ.

## Conclusion

The jowl, a stigmatizing sign of aging in humans, results from aging of the constantly moving subcutaneous tissues and skin overlying the mandible. The presence of this mobile tissue in humans results from the evolution of the mouth and its mimetic muscles. The mandibular ligament proper is not osteocutaneous nor stout, as it does not have a stout subcutaneous component, nor does it constitute the anterior border of the jowl. It is present only in the deep plane and is a different name for the mandibular attachment of the mimetic muscles (platysma, DLI and DAO) and is present under the anterior half of the jowl. The labiomandibular crease is not formed by a ligament, instead it relates to the insertion of the lower lip depressors into the dermis. The MMN is in direct proximity of the mandibular ligament, and release of the mandibular ligament in the deep plane is contraindicated. Release of a “mandibular ligament” at the anterior border of the jowl is a misconception.

## Supplementary Information

Below is the link to the electronic supplementary material.

Supplementary file1 (DOCX 17 kb)

Supplementary file2 (MP4 44293 kb)

Supplementary file3 (MP4 55003 kb)

Supplementary file4 (MP4 24910 kb)

## Declarations

### Presented at

ISAPS 2021 World Congress in Vienna, Austria.
