# Supplementary material for: The Surgical Anatomy of the Jowl and the Mandibular Ligament Reassessed
Source: Aesthetic Plast Surg. 2022 Sep 1;47(1):170–80. doi: 10.1007/s00266-022-02996-3 (PMC9944027; doi:10.1007/s00266-022-02996-3)
Supplement: Supplementary file 1 — Supplementary file1 (DOCX 17 kb) [file 266_2022_2996_MOESM1_ESM.docx]

**Video 1**

The effect of opening the mouth on the relationship of the mandible with the platysma, where the platysma and mandible are attached, at the mandibular ligament (white circle marks the end of the ligament) they move en-bloc, whereas posterior to the mandibular ligament, the mandible glides under the platysma.

**Video 2**

Demonstration of the mandibular ligament, or common attachment of the platysma, DLI and DAO to the mandible. Observe how the platysma and DLI are part of the same muscle sheet and therefore do not overlap. Elevation of the superficially located DAO reveals the continuation of the platysma as the DLI. The main marginal mandibular nerve (MMN) is seen in close proximity to the mandibular ligament.

**Video 3**

Demonstration on how a mandibular ligament can appear to be present in the subcutaneous layer when dissecting in the deep subcutaneous plane. A deep subcutaneous dissection cuts the retinacula cutis at their base (trunks). Any movement of the flap is therefore a reflection of the underlying layer. Posterior to the mandibular ligament gliding of the platysma over the underlying deep fascia allows significant movement of the flap. However, over the mandibular ligament the platysma is fixed. The result is that it can appear that the skin is tethered in this area, while in fact the skin is tethered in all areas. It is the platysma which is tethered in this specific area.
